# Anti-Asialo GM1 NK Cell Depleting Antibody Does Not Alter the Development of Bleomycin Induced Pulmonary Fibrosis

**DOI:** 10.1371/journal.pone.0099350

**Published:** 2014-06-12

**Authors:** Justin Monnier, Brian A. Zabel

**Affiliations:** 1 Department of Pathology, Stanford University School of Medicine, Stanford, California, United States of America; 2 Palo Alto Institute for Research and Education, Department of Veterans Affairs Palo Alto Health Care System, Palo Alto, California, United States of America; French National Centre for Scientific Research, France

## Abstract

Despite circumstantial evidence postulating a protective role for NK cells in many fibrotic conditions, their contribution to the development of pulmonary fibrosis has yet to be tested. Lung-migrating NK cells are thought to attenuate the development of bleomycin induced pulmonary fibrosis (BIPF) by providing anti-fibrotic mediators and cytokines, such as IFN-γ. If true, we reasoned that depletion of NK cells during experimentally-induced fibrotic disease would lead to exacerbated fibrosis. To test this, we treated mice with NK cell-depleting antisera (anti-asialo GM1) and evaluated lung inflammation and fibrosis in the BIPF model. While NK cell infiltration into the airways was maximal at day 10 after bleomycin injection, NK cells represented a minor portion (1–3%) of the total leukocytes in BAL fluid. Anti-asialo GM1 significantly abrogated NK cell numbers over the course of the disease. Depletion of NK cells with anti-asialo GM1 before and throughout the BIPF model, or during just the fibrotic phase did not alter fibrosis development or affect the levels of any of the pro-inflammatory/pro-fibrotic cytokines measured (IL-1β, IL-17, IFN-γ, TGF-β and TNF-α). In addition, adoptively transferred NK cells, which were detectable systemically and in the airways throughout BIPF, failed to impact lung fibrosis. These findings indicate that NK cells likely do not play an essential protective role in controlling pulmonary fibrosis development.

## Introduction

Pulmonary fibrosis is a progressive lung disease characterized by the irreversible formation of scar tissue throughout the lungs, which ultimately leads to respiratory failure [1], [2], [3]. The etiologies of pulmonary fibrosis are diverse (including prescribed exposure to the chemotherapeutic bleomycin) and in some cases the causes are unknown (Idiopathic pulmonary fibrosis) [2]. Pulmonary fibrosis is currently irreversible, and patients only have 2–6 years' life expectancy after diagnosis [3]. Much of our understanding of the molecular and cellular mechanisms governing pulmonary fibrosis is derived from in vivo mouse studies using the BIPF model, in which lung fibrosis is induced with a single administration of bleomycin [4].

Development of BIPF involves a complex ballet between the coagulation cascade, inflammatory response, and lung tissue remodeling [5]. Over the years a strong effort has been devoted to clarifying the immunological response during BIPF. As a result the list of leukocytes (neutrophils, lymphocytes, macrophages, eosinophils) and secreted cytokines and growth factors (TGF-β, PDGF, TNF-α, IFN-γ, IL-17, IL-1, IL-13…) involved in the progression of pulmonary fibrosis is extensive [5]. However, not all of the inflammatory cells that migrate to the lungs and airways during BIPF are thought to be pathogenic. NK cells, for example have been hypothesized to dampen pulmonary fibrosis [6]. NK cells may induce anti-fibrotic signals in liver and in lung through two independent mechanisms: 1) contact dependent interactions where NK cells can block liver fibrosis by directly killing activated liver collagen producing fibroblasts or 2) through the release of soluble anti-fibrotic mediators such as putative anti-fibrotic cytokine IFN-γ [7], [8]. In pulmonary fibrosis, NK cells are thought to provide protection against bleomycin induced injury through the production IFN-γ, which is believed to counteract the pro-fibrotic activities of TGF-β [6], [9], [10].

To decipher the contribution of NK cells to the development of pulmonary fibrosis, we opted to systemically deplete NK cell over the course of the disease using an antibody based approach. Systemic depletion of NK cells was achieved using the anti-asialo GM1 antibody, which was injected at different times during the BIPF model, both immediately before and throughout the acute inflammatory phase (days 1–10) or before the fibrotic phase (days 10–21) of disease, or only during the fibrotic phase. Anti-asialo GM1 is a rabbit polyclonal antibody from that reacts with a neutral glycosphingolipid expressed on the surface of numerous hematopoietic cells including NK, NKT, CD8+T, γδT, some CD4+T cells, macrophages, eosinophils and basophils [11], [12], [13], [14], [15]. Nevertheless, anti-asialo GM1 only effectively eliminates NK cells and basophils in vivo [12], [16]. Other less discriminating NK cell-depleting antibodies exist such as anti-NK1.1, but it also depletes NKT cells, which are significant producers of IFN-γ during BIPF [17]. There are also genetically modified mice with NK cell deficiencies, such as Beige and Stat5 (f/f) Ncr1-iCreTg mice. Unfortunately neither of these models is ideal for assessing the role of NK cells in BIPF. While Beige mice completely lack NK cells, they are also deficient in cytotoxic T cells and have impaired neutrophil activity, which complicates data interpretation. On the other hand, while NK cell depletion in Stat5(f/f) Ncr1-iCreTg mice is selective, it is not complete, with residual NK numbers comparable to WT mice treated with anti-asialo GM1 antibody [18], [19]. Therefore, anti-asialo GM1 antibody is one of the most precise tools available to specifically eliminate NK cells in vivo. We tested two different depletion strategies to 1) evaluate the overall contribution of NK cells during the initial inflammatory phase (IFN-γ producing phase) and/or 2) to evaluate the role of NK cells during the fibrotic phase of the disease (potential fibroblast-killing phase). Our results show that while NK cells were effectively depleted when anti-asialo GM1 was administered in either mode, the development of BIPF remained unaltered.

To complement the depletion experiments, we also assessed the impact of adoptively-transferred NK cells in the pathogenesis of BIPF. Although adoptive transfer of NKT cells protected against BIPF [20], in our experiments supplemental NK cells had no impact on the course of disease. Thus the aggregate of our data indicate that NK cells do not play a central role in regulating pulmonary fibrosis.

## Materials and Methods

All animal studies and procedures were approved by the Institutional Animal Use and Care Committee at the Veterans Affairs Palo Alto Health Care System (animal welfare assurance number A3088-01; AAALAC-accredited facility).

### Animals

6-10 week old female Balb/c (CD45.2+) and Balb/cBYJ (CD45.2+) mice were obtained from (Jackson Laboratory, Bar Harbor, Maine, USA). All mice were housed in a specific pathogen free facility and screened regularly for pathogens.

### Bleomycin Administration

Mice were anesthetized by isoflurane inhalation before intranasal injection of 50 µl sterile saline solution containing 6 U/kg (7.5 mg/kg) bleomycin (Sigma, St. Louis, MO, USA). The mice were monitored and weighed daily for the duration of the experiment. At the indicated time points animals were euthanized by CO2 asphyxiation.

### In vivo NK cell depletion

Mice were injected i.p. with 100 µl of anti-asialo GM1 or control rabbit sera (Wako Chemicals, Richmond, VA, USA) diluted 1∶10 in PBS on the days indicated.

### Bronchoalveolar Lavage (BAL) Fluid Collection and Analysis

After mice were euthanized, a blunt needle was inserted in the exposed trachea. The airway of the mice was washed three times with 1 ml PBS. The recovered fluid was centrifuged and the recovered leukocytes in the BAL fluid were directly stained with surface markers for T cells (TCR-β), neutrophils (Ly6G), and NK cells (DX5), B-cells (CD19), and macrophages (F4/80). Antibodies that recognize allotypic markers CD45.1 and CD45.2 were used to track adoptively-transferred NK cells. Cell-free BAL fluid was analyzed for soluble collagen content and cytokine concentrations.

### Lung Homogenate Processing

At the indicated time points, mice were euthanized and the lungs were perfused using 10 ml PBS by cardiac puncture. The left lung was collected, weighed and homogenized in 10 µl of PBS + protease inhibitors/10 mg lung tissue (Roche, Indianapolis, Indiana, USA). Lung homogenates were then centrifuged for 5 min at 3000 RPM at 4C, and the supernatants were collected and stored at-20C for further experimentation.

### Blood and Spleen Leukocyte Isolation

Blood was collected by cardiac puncture after euthanasia and directly mixed with 5 ml PBS without Ca2+/Mg2+ supplemented with 4 mM EDTA to prevent clotting. An equal volume of dextran-T-500 was added, the solution gently mixed by inversion, and incubated at 37°C for 45 min. The supernatant was collected and centrifuged and incubated with 2 ml red blood cell lysis buffer (Sigma, St. Louis, MO, USA). The pelleted white blood cells were then stained and analyzed by flow cytometry. Spleens were collected, and crushed over a 40 um cell strainer with 10 ml of cold PBS. After centrifugation, the supernatant was discarded and red blood cells were lysed using a red cell lysis buffer (Sigma, St. Louis, MO, USA). The leukocytes were used for flow cytometry.

### Histology and Collagen Quantification

Lungs were perfused with 10 ml cold PBS. The lungs were then harvested, and the right lung lobes were placed in 10% formalin and embedded in paraffin for sectioning (Histo-tech lab, Hayward, CA, USA). Mason's trichrome was used to stain collagen and evaluate fibrosis. The degree of fibrosis in each lung section was quantified using the Ashcroft scoring system, which provides a scale for grading the severity of lung fibrosis that ranges from 0 (normal lung) to 8 (total fibrous obliteration of the field) [21]. Lung collagen content was measured by the hydroxyproline assay according to the manufacturer's instructions (Biovision, Milpitas, CA, USA). BAL fluid collagen was measured using the Sircol collagen dye binding assay, according to the manufacturer's instructions (Biocolor, Carrickfergus, United Kingdom).

### ELISA

mTGF-β (R&D system, Minneapolis, MN), mIL-17A (biolegend, San Diego, CA), mTNF-α (BD biosciences, San Jose, CA, USA), mIFN-γ (BD biosciences), mIL-1β (BD biosciences) concentrations in BAL fluid or in lung homogenates were measured by ELISA according to the manufacturer's instructions.

### Flow Cytometry

A total of 0.5 million cells were used for each staining. Cells were incubated with directly-labeled antibodies at 4°C for 30 min in 100 µl in PBS/2% FBS/2% mouse serum. Cells were washed and centrifuged for 3 min at 2000 rpm, re-suspended and fixed in 200 µl of PBS/1%PFA (paraformaldehyde) and were analyzed using a FACS-Calibur (BD Biosciences, San Jose, CA, USA).

### Adoptive Transfer of NK Cells

NK cells were isolated from the spleens of WT 6–8 week old female balb/c mice using untouched NK cell magnetic bead purification kit (NK Cell Isolation Kit II, Miltenyi Biotech, San Diego, CA, USA). Purification was monitored after each column passage by flow cytometry, and NK cells were used for transfer once the purity of the sample reached >80%. After purification NK cells were re-suspended in saline (10 million cells/ml), and 100 µl (1 million cells) or saline alone was injected i.v. (tail vein) into syngeneic balb/c female mice. The number of transferred NK cells injected was based on previous reports that demonstrated potent biologicals effect of 0.5–1 million transferred NK cells in a variety of lung pathologies [22], [23], [24].

### Statistics

Evaluation of significance was performed using Student's *t*-test and ANOVA. Statistical tests were calculated using the Instat statistical program (Graphpad, La Jolla, CA, USA), and graphs were plotted using Prism graphing software (Graphpad, La Jolla, CA, USA). Data are expressed as mean ± SD or SEM as indicated, and p values less than 0.05 were considered to be significant.

## Results

### NK cells represent a small portion of the total leukocytes in BAL fluid

**Figure 1:**
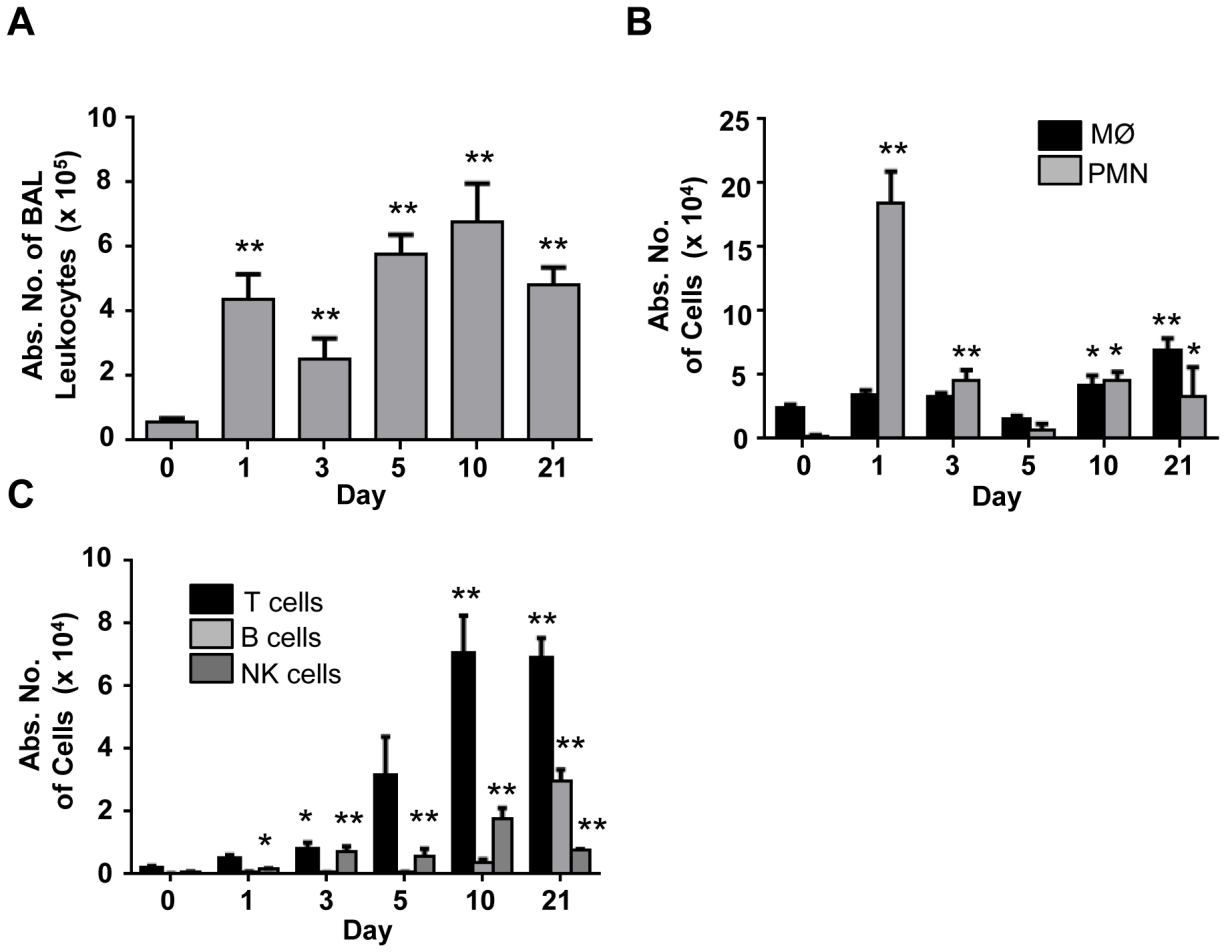
Kinetic analysis of leukocyte migration in the airways in bleomycin treated mice. Leukocytes from BAL fluid were collected on day 0, 1, 3, 5, 10 and 21 after intra-nasal injection of bleomycin (7.5 mg/kg) in WT balb/c mice. **A**) The absolute number of leukocytes in BAL fluid was determined by flow cytometry at the indicated time points. **B**) The number of macrophages and neutrophils were determined by flow cytometry using specific antibodies against macrophages (F4/80) and neutrophils (Ly6G). **C**) The number of T cells (TCRβ+), B cells (CD19+), and NK cells (DX5+TCRβ-) were measured in BAL fluid by flow cytometry. n = 3–8 mice per time point. *p<0.05, ** p<0.01, by ANOVA followed by t-test, comparing indicated time points post-bleomycin injection to t = 0.

Leukocyte subsets infiltrated the bronchoalveolar space at different rates and magnitudes during BIPF. The total number of recruited leukocytes remained significantly elevated from day 1–21 following bleomycin administration (Fig. 1A). Neutrophil numbers spiked in BAL fluid on day 1 but rapidly decrease by day 3 (Fig. 1B). Macrophages gradually accumulated through day 21. T cell and B cell numbers remained low during the first 7–10 days, and reached their apex on day 21 (Fig. 1C). NK cells comprised 1–3% of total BAL leukocytes at any time point evaluated, including day 0 (not shown, no significant differences). Numerically, NK cells comprised the smallest lymphocyte population in BAL fluid, with maximal accumulation on day 10 (Fig. 1C).

### Anti-asialo GM1 antibody treatment specifically and rapidly depletes NK cells

**Figure 2:**
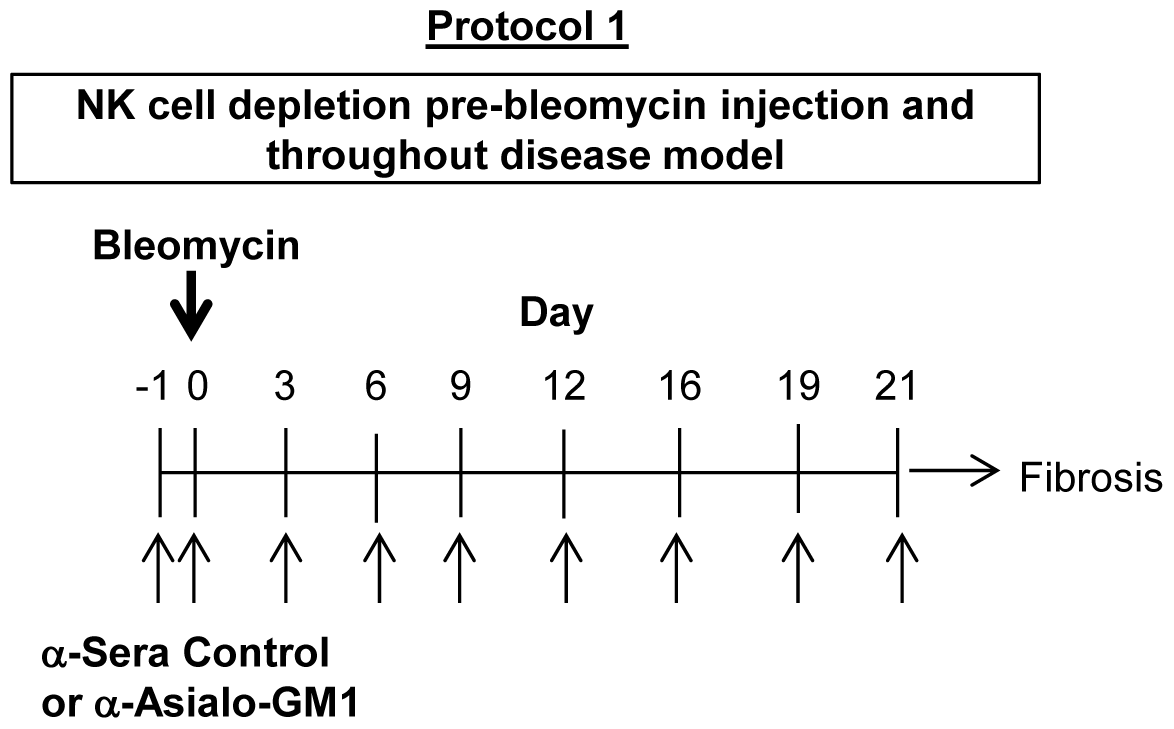
NK cell depleting anti-asialo GM1 antibody treatment protocol during bleomycin induced pulmonary fibrosis. **Protocol 1**: depicts the treatment of mice with either control sera or anti-asialo GM1 antibody to deplete NK cells during BIPF. Mice were given eight injections of depleting antibody or control sera at the indicated times before and after i.n. bleomycin dosing.

**Figure 3:**
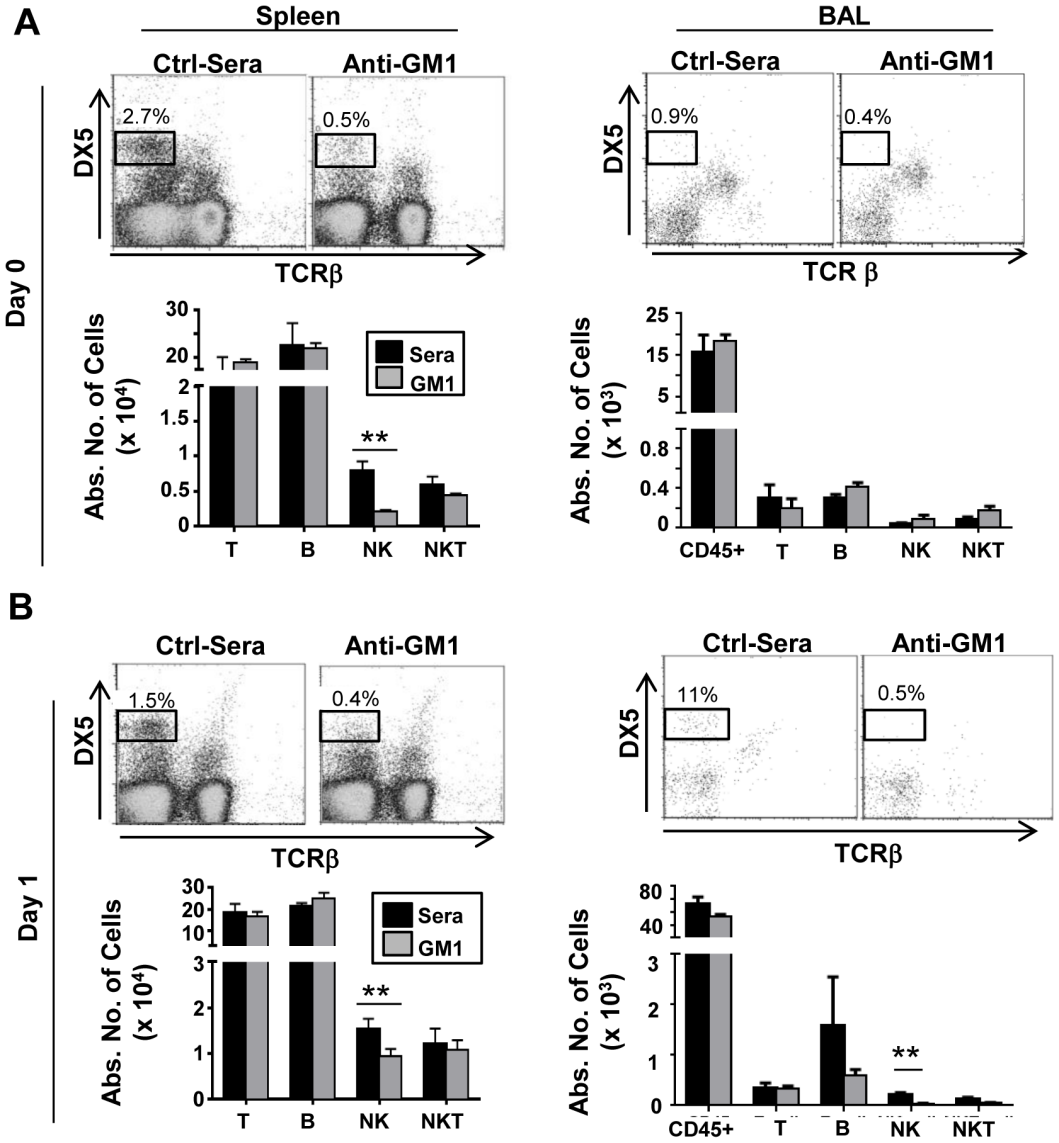
Pre-treatment with anti-asialo GM1 depletes NK cells in spleen and airways before bleomycin injection. Mice were injected with either anti-asialo GM1 or control rabbit sera at −24 h and −1 h before bleomycin administration (**A**) At day 0, before bleomycin injection, and (**B**) day 1 after bleomycin injection leukocytes were isolated from spleen and BAL fluid, stained and analyzed by flow cytometry. Leukocytes were identified by CD45 expression, and percentages and absolute numbers of T cells (TCRβ+), B cells (CD19+), NK cells (DX5+TCRβ-) and NKT cells (DX5+TCRβ+) were determined. In the BAL fluid analysis in (**B**), a single value in the quantification of absolute T cell numbers (data point: 2,453 T cells) in the sera-control group was determined by Dixon's Q test to be an outlier (>95% confidence), and was therefore excluded from subsequent analysis. Mean ± SEM of n = 4 mice per group. *p<0.05, ** p<0.01 by 2 way ANOVA followed by *t*-test.

Anti-asialo GM1 antibody or control rabbit serum was injected in mice −24 h and −1 h before bleomycin injection to deplete NK cells (Fig. 2). To determine the efficiency of NK cell depletion in the absence of bleomycin challenge, on day 0 we collected BAL fluid and spleens from either control sera or anti-asialo GM1 pre-treated mice and evaluated the absolute number of leukocytes by flow cytometry. Anti-asialo GM1 treatment significantly depleted splenic NK cells, but did not significantly alter the number of the few detectable NK cells in BAL (Fig. 3A). Anti-asialo GM1 treatment did not affect the number of T, B and NKT cells in the spleen or in the BAL fluid, demonstrating NK-selective depletion specificity (Fig. 3A).

To determine the efficiency of NK cell depletion one day post-bleomycin challenge, on day 1 we collected BAL fluid and spleens from either control sera or anti-asialo GM1 treated mice and evaluated the absolute number of leukocytes by flow cytometry. Anti-asialo GM1 treatment significantly depleted splenic and BAL fluid NK cells, but had no effect on T, B and NKT cells numbers (Fig. 3B). These studies therefore validated the capability of anti-asialo GM1 to significantly and specifically abrogate systemic and airway-recruited NK cells in BIPF.

### Sustained anti-asialo GM1 treatment maintains systemic and airway-specific NK cell suppression during BIPF

**Figure 4:**
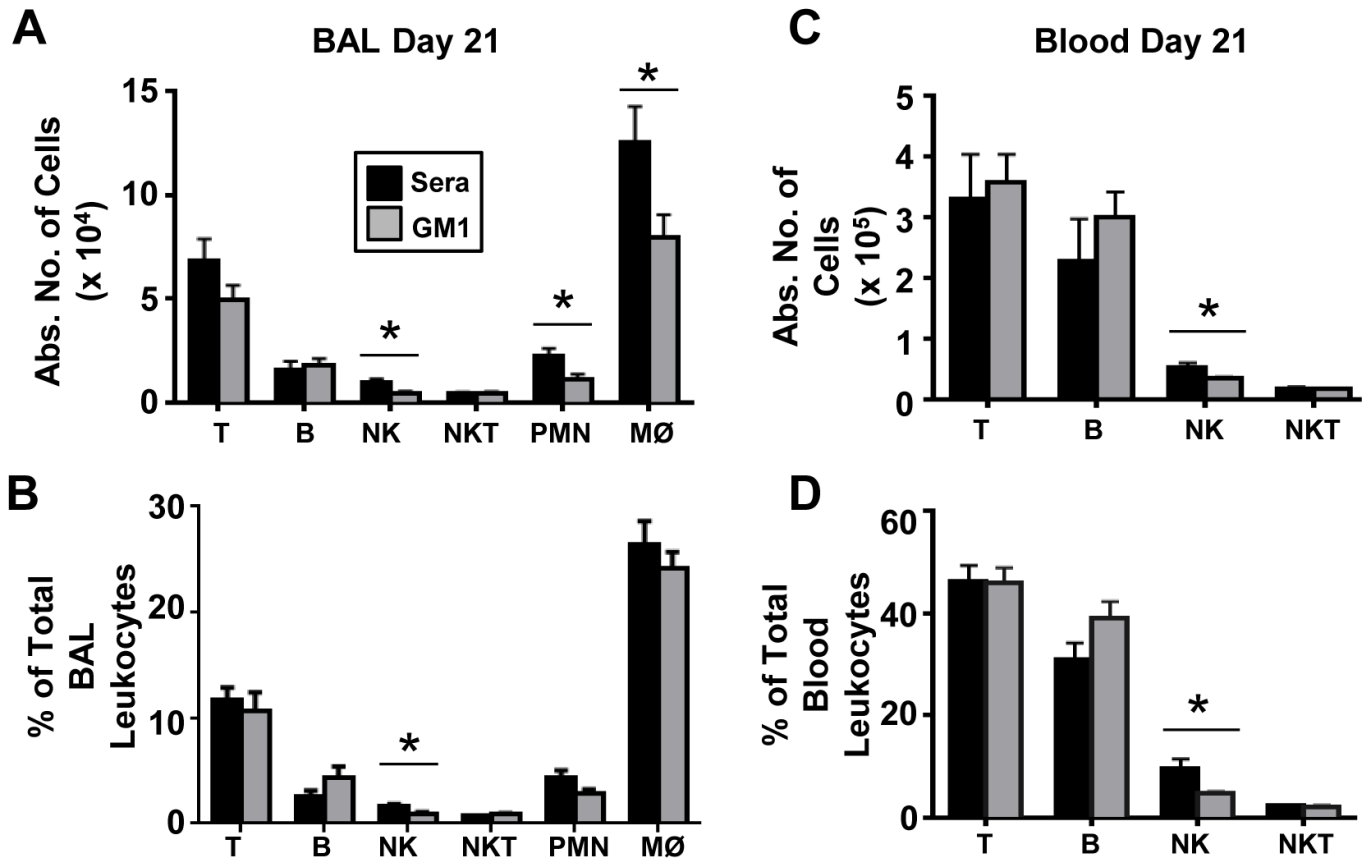
Anti-asialo GM1 treated mice maintain depleted levels of NK cells at day 21 after bleomycin injection. At day 21 after bleomycin injection, leukocytes were isolated from BAL fluid (**A, B**) or blood (**C, D**) and stained with specific antibodies against NK cells (DX5); T cells (TCRβ), B cells (CD19) and NKT (TCRβ+DX5+), neutrophils (Ly6G), and macrophages (F4/80) and analyzed by flow cytometry. Mean ± SEM of n = 8–10 mice per group. *p<0.05, ** p<0.01 by 2 way ANOVA followed by *t*-test.

Mice were pre-treated twice with either control sera or anti-asialo GM1 antibody in the 24 hours preceding bleomycin injection, and thereafter mice were treated every 3–4 days to maintain continuous systemic suppression of NK cells (Fig. 2). On day 21 post-bleomycin challenge, BAL fluid and blood were collected. Anti-asialo GM1 treatment significantly depleted BAL fluid and blood NK cells (measured as both absolute numbers and percentages), but had no effect on T, B or NKT cells (Fig. 4). While there was also a significant reduction in the total number of airway neutrophils and macrophages (Fig. 4A), this difference did not alter their percentages as airway infiltrating leukocytes (Fig. 4B).

### Depletion of NK cells does not affect the development of fibrosis

**Figure 5:**
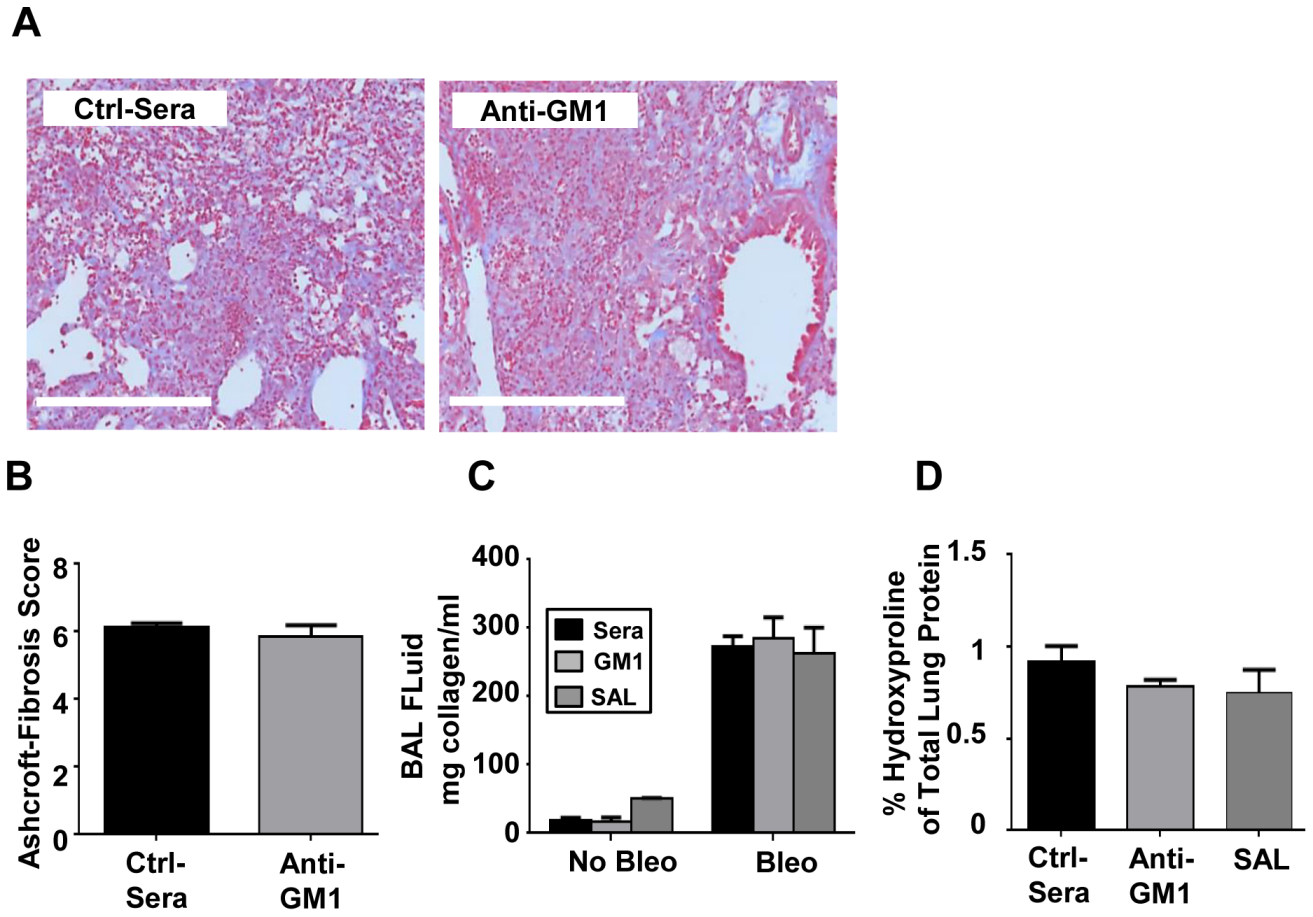
Anti-asialo GM1 treatment does not alter development of BIPF. (**A**) On day 21 after bleomycin treatment, lungs from control sera or anti-asialo GM1 treated mice were isolated, embedded in formalin, sectioned and stained with Mason's Trichrome for visualization of collagen deposition. Quantification of fibrosis score was performed using the Ashcroft scoring system. Mean ± SEM of n = 5 mice per group. (**B**) Soluble collagen levels in BAL fluid were measured by Sircol assay from mice treated with saline (SAL), control sera, or anti-asialo-GM1. (**C**) Lung collagen content was measured in lung homogenates on day 21 post bleomycin administration by hydroxyproline assay, and the amount of hydroyxproline was calculated as a percent of total lung protein. Mean ± SEM of n = 8–10 mice per group.

**Figure 6:**
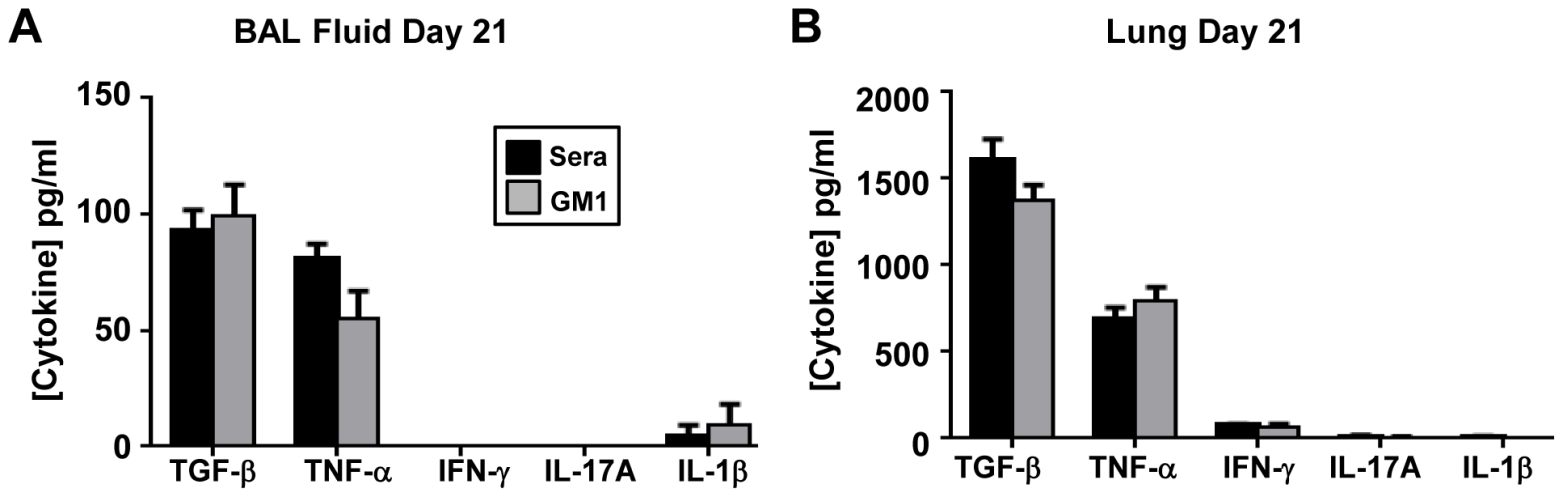
BAL fluid and lung cytokine levels on day 21 post-bleomycin injection. IL-1β, TGF-β, TNF-α, IFN-γ, IL-17A levels were measured by ELISA in (**A**) BAL fluid or in (**B**) lung homogenates from mice treated with control sera control or anti-asialo GM1. Mean ±SEM of n = 7–10 mice per group.

We next asked if sustained NK cell depletion altered the disease course of BIPF. Twenty-one days post-bleomycin challenge the lung tissue from anti-asialo GM1 antibody vs. control sera treated mice was analyzed for collagen content by histology. In line with other reports, bleomycin induced key histopathological features of fibrosis, including moderate thickening of alveolar and bronchoalveolar walls, obvious damage to lung architecture, formation of fibrous bands and small fibrous masses [21]. There was no difference in histopathological features or collagen deposition in lung sections between control sera and anti-asialo GM1 treated mice (Fig. 5A). We next evaluated the soluble collagen content in BAL fluid by Sircol assay, a complementary biochemical method of quantifying fibrosis [25]. Collagen concentrations in the BAL fluid and lung homogenates were not significantly different among saline, control sera, or GM1 treated mice (Fig. 5B, C). Consistent with other reports, bleomycin-challenged mice lost a significant amount of weight, although there were no differences between treatment groups (Fig. 5D). We next asked if there were any differences in the concentrations of key cytokines (IL-1β, IL-17A, IFN-γ, TGF-β and TNF-α) known to play a role in inflammation/fibrosis during BIPF. There were no differences in BAL fluid or lung homogenate cytokine levels between treatment groups by ELISA (Fig. 6A, B). Thus prolonged abrogation of NK cells during the acute inflammatory phase and fibrotic phase of BIPF did not alter the levels of key cytokines or affect collagen deposition and fibrotic scarring of the lungs.

### Depletion of NK cells during the fibrotic phase of BIPF does not alter fibrosis development

**Figure 7:**
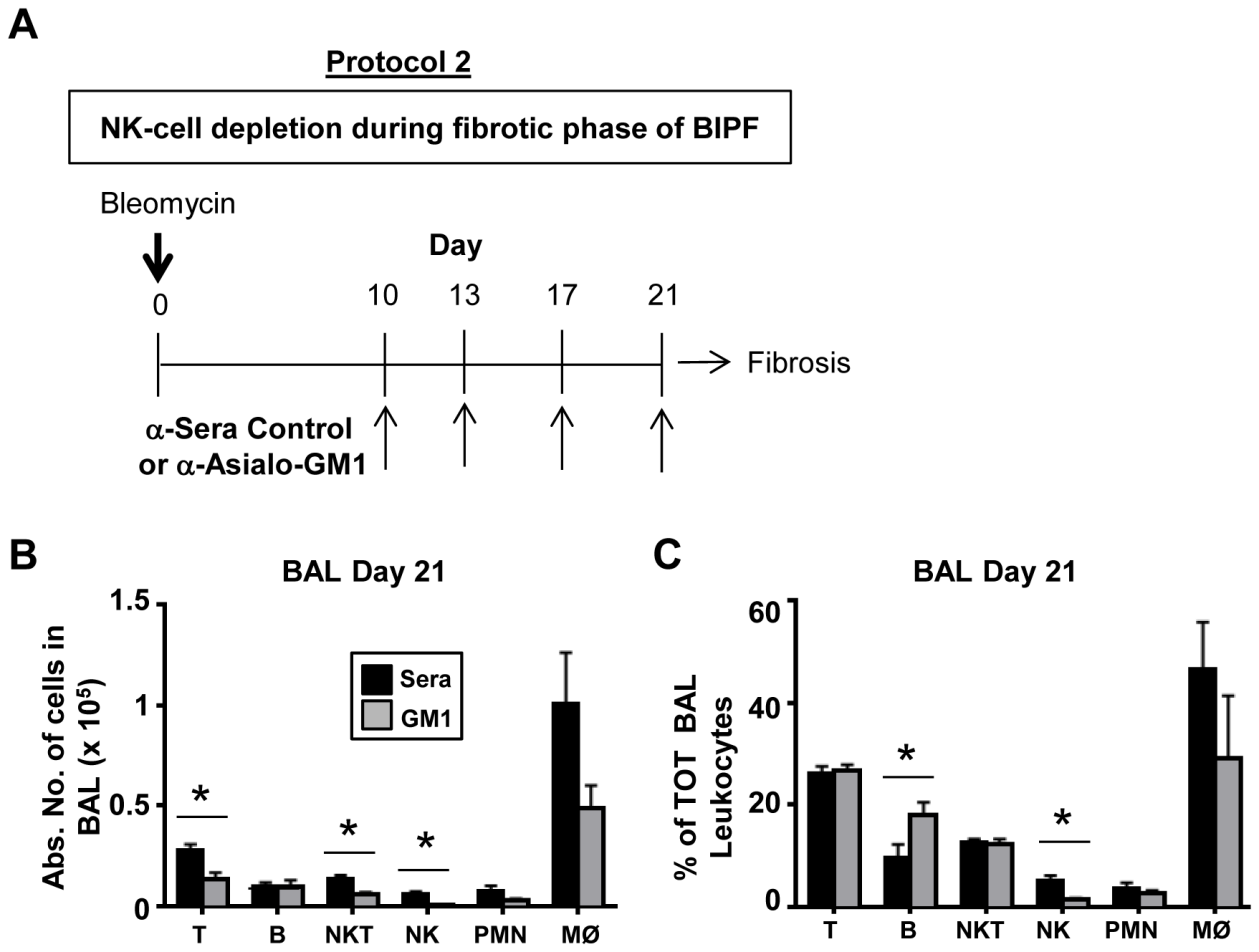
Anti-asialo GM1 treatment during the fibrotic phase of BIPF results in NK cell depletion. **A**) **Protocol 2**: depicts the treatment of mice with either isotype control or anti-asialo GM1 antibody to deplete NK cells during BIPF. Mice were given four injections of depleting antibody or control sera control at the indicated times after i.n. bleomycin dosing, starting on day 10. On day 21 after bleomycin injection, leukocytes were isolated from BAL fluid and stained with specific antibodies against NK cells (DX5) T-cells (TCRβ), B-cells (CD19) and NKT (TCRβ+, DX5+), neutrophils (Ly6G), macrophages (F4/80) and analyzed by flow cytometry. The results are either expressed as absolute number of cells (**B**) or as a percentage of the total leukocytes (**C**). Mean ±SEM of n = 5 mice per group. *p<0.05, ** p<0.01 by *t*-test.

**Figure 8:**
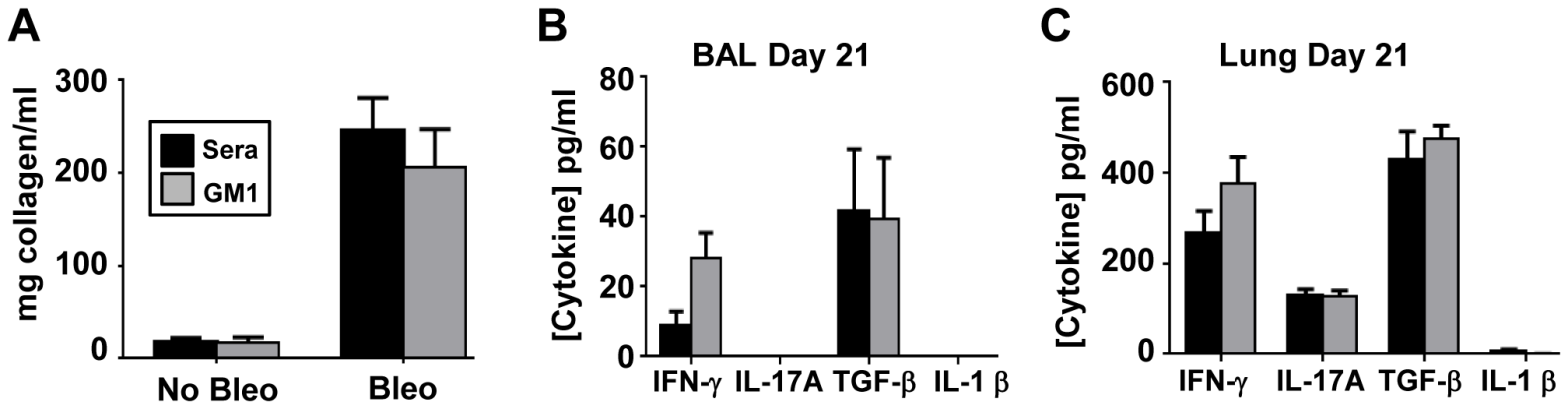
Anti-asialo GM1 treatment during the fibrotic phase of BIPF does not alter fibrosis development. (**A**) BAL collagen concentration was measured using the Sircol assay. IL-1β, TGF-β, IFN-γ, IL-17A levels were measured by ELISA in (**C**) BAL fluid or in (**D**) lung homogenates from mice treated with control sera control or anti-asialo GM1. Mean ± SEM of n = 5 mice per group.

We next asked if NK cell depletion by anti-asialo GM1 isolated temporally to the fibrotic phase of BIPF would alter or exacerbate fibrosis. The initiation of the fibrotic phase of BIPF begins on day 10 post-bleomycin challenge [5], which coincides with peak NK cell migration into the airways (Fig. 1C). We therefore began treating BIPF mice with anti-asialo GM1 or control sera on day 10 and every 3–4 days after until day 21, as depicted in Fig. 7A. On day 21 the mice were sacrificed and leukocytes were isolated from BAL and blood, stained, and analyzed by flow cytometry. The absolute number of NK cells and their percent of total leukocytes were significantly lower in BAL fluid from anti-asialo GM1-treated mice vs. controls, confirming the efficacy of NK-depletion (Fig.7B, C). The effect of anti-asialo GM1 was largely selective for NK cells, as there were no differences in T cell, B cell, or neutrophil numbers or percentages in BAL fluid between treatment groups (Fig. 7B, C). There was a significant reduction in the absolute number of airway NKT cells; however, this was not reflected in their percent of total leukocytes in anti-asialo GM1 treated BIPF mice (Fig. 7B, C). We next assessed the collagen content in BAL fluid by Sircol assay as a surrogate biomarker of lung fibrosis. There were no differences in collagen concentrations in the BAL fluid in mice treated with control sera or anti-asialo GM1 during the fibrotic phase of BIPF (Fig. 8A), nor were there differences in weight loss between treatment groups (Fig. 8B). There were also no differences in BAL fluid or lung homogenate IL-1β, IL-17A, IFN-γ, and TGF-β levels between treatment groups by ELISA (Fig. 8C, D). Thus depletion of NK cells limited to the fibrotic phase of BIPF did not alter the levels of key cytokines or ultimately affect collagen deposition.

### Adoptive transfer of NK cells does not alter fibrosis development

**Figure 9:**
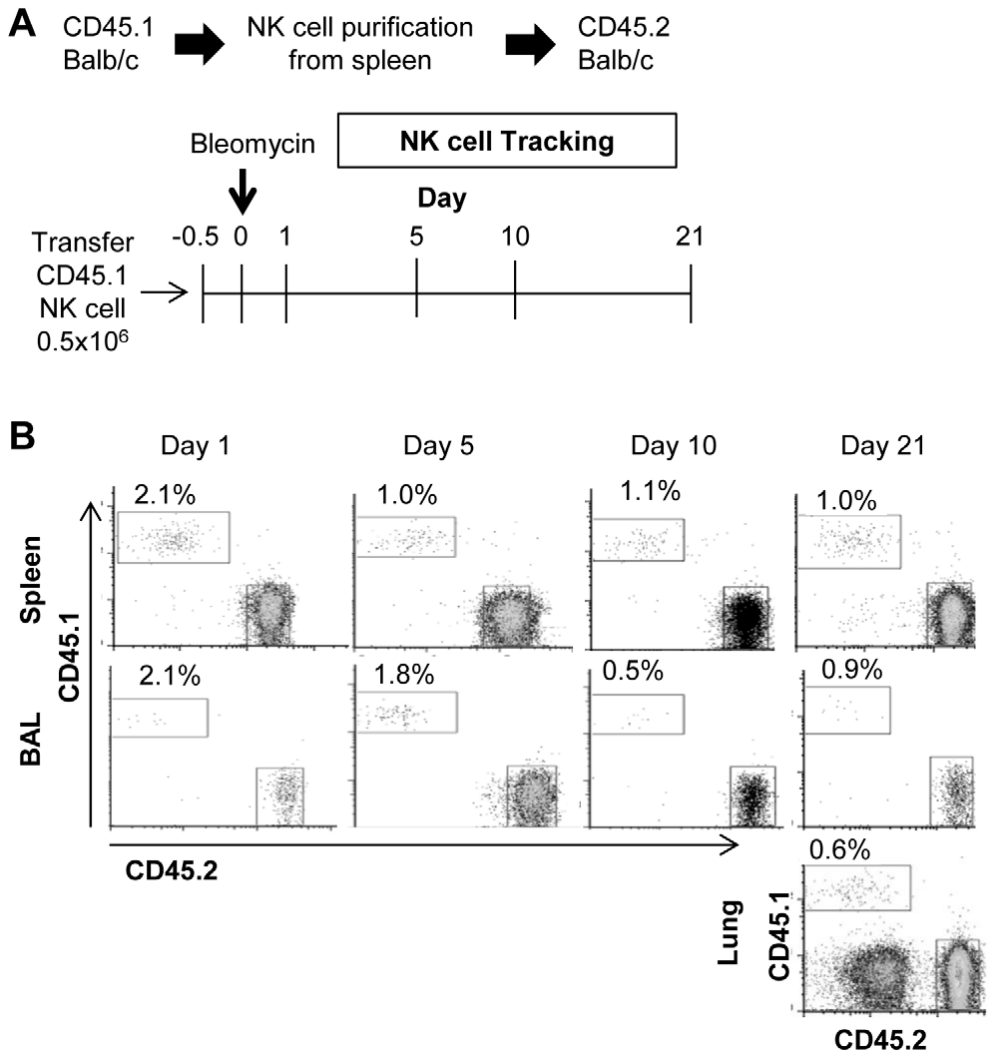
Distribution and abundance of adoptively transferred CD45.1+ NK cells into CD45.2+ recipients during BIPF. (**A**) After NK cell purification by magnetic beads, 0.5 million CD45.1+ NK cells were injected i.v. into CD45.2+ recipients. Mice were injected intranasally with bleomycin (7.5 mg/kg) 12 h post-cell transfer (**B**) At the indicated time points, spleen, BAL fluid and lungs were harvested, and the donor (CD45.1+) and recipient (CD45.2+) NK cells (DX5+CD3-) were quantified by flow cytometry.

**Figure 10:**
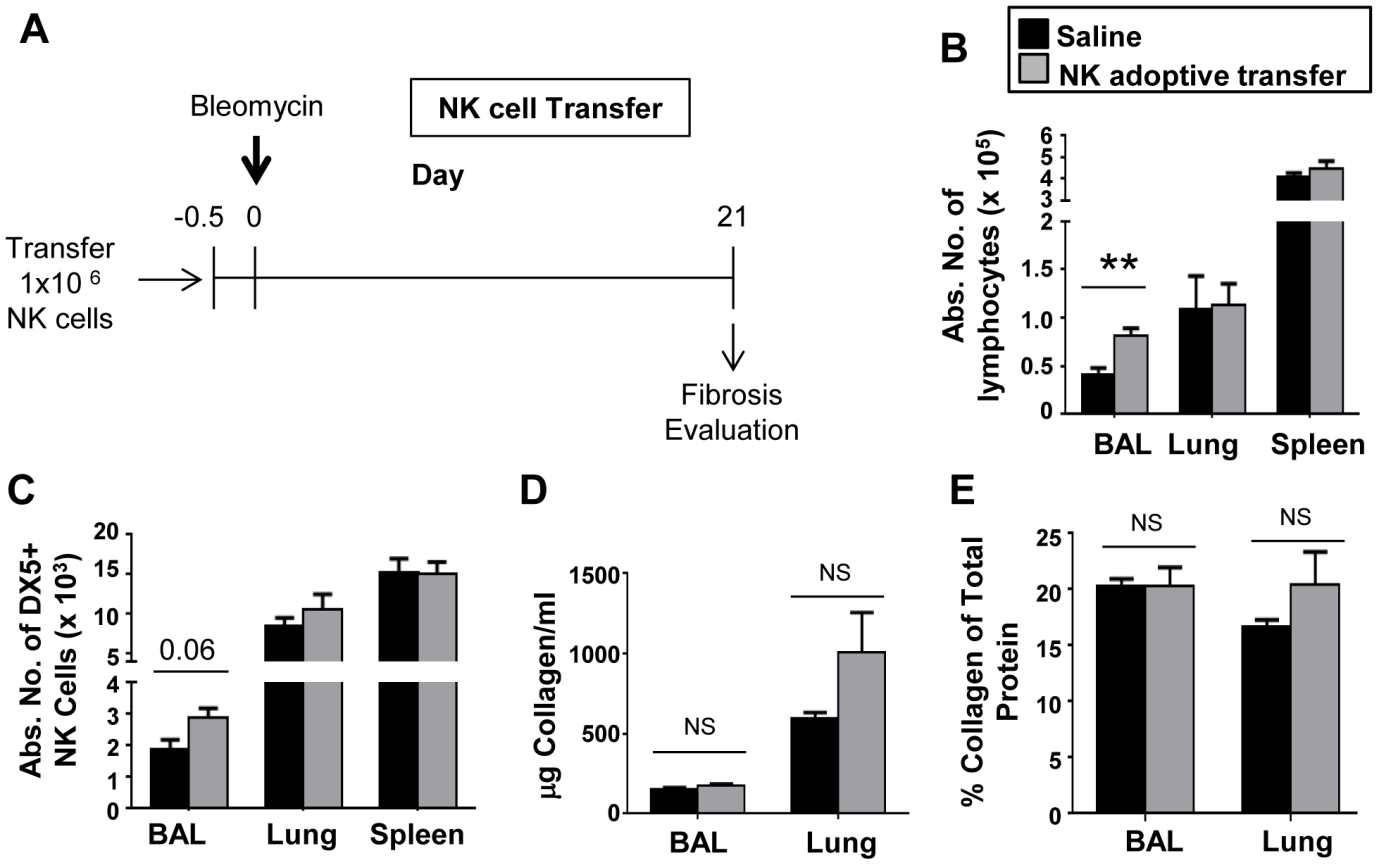
Adoptive transfer of NK cells does not alter pulmonary fibrosis in BIPF. (**A**) Recipient mice were injected with 1 million purified donor NK cells 12 h prior to bleomycin injection. (**B, C**) On day 21 leukocytes from BAL fluid, lungs, and spleen were collected, quantified, and stained with specific antibodies for leukocytes (CD45), NK cells (DX5), and T cells (TCRβ) and analyzed by flow cytometry. (**D, E**) Soluble collagen levels in BAL fluid and lung homogenates were measured by Sircol assay; total protein content was measured by BCA assay. Mean ± SEM of n = 5 mice per group; ** p<0.01 by *t*-test.

To complement our depletion studies, we also asked if NK cell supplementation could impact disease progression in BIPF. First we assessed the survival and distribution of transferred NK cells in the context of BIPF. We injected purified CD45.1+ NK cells into CD45.2 balb/c congenic recipients and tracked their distribution in airways, spleen, and lung parenchyma over time following bleomycin injection (Fig. 9A). At every time point investigated (day 1, 5, 10 and 21) CD45.1 NK cells were detected in BAL fluid, spleen or lung (Fig. 9B). These results imply that transferred NK cells survive in vivo and traffic to the relevant anatomic sites to potentially impact disease during BIPF. We next asked if transferred NK cells had any impact on lung fibrosis on day 21 following bleomycin injection (Fig. 10A). Adoptive transfer of 1 million NK cells resulted in a significant increase in airway lymphocytes but had no effect on total lymphocyte numbers in the spleen or lung parenchyma (Figure 10B). There was an increase in DX5+ NK cells in the BAL, although it did not reach statistical significance (Fig. 10C). Finally, adoptively transferred NK cells had no effect on lung fibrosis as determined by either total collagen quantification in the BAL and lungs (Fig. 10D), or as a percent of collagen per total protein (Fig. 10E).

## Discussion

Depletion of NK cells by anti-asialo GM1 antibody is a commonly used approach to study the contribution of NK cells to a wide range of immune-related pathophysiological processes. However, this is the first study to our knowledge that has investigated the use of anti-asialo GM1 in depleting NK cells during BIPF. Here we show that treatment of mice with anti-asialo GM1 antibody during BIPF results in significant systemic and airway NK cell abrogation but ultimately does not alter lung fibrosis.

Before performing the in vivo NK cell depletion experiments, we sought to fully evaluate the kinetic profile of NK cell migration into the airways during BIPF. Consistent with another report, the acute inflammatory phase of BIPF was characterized by a large infiltration of neutrophils [5]. As the disease evolved towards fibrosis, there was an increase in airway-infiltrating macrophages, T cells and B cells, with T cells and macrophages being the predominant cell types on day 21. Interestingly, NK cells were present in the airways over the entire course of disease, although they represented a minor fraction of the total leukocyte population (1–3%) on any given day. NK cells migrated into the airways on day 1 after bleomycin injection; their numbers peaked on day 10, and a significant number of NK cells were also present on day 21.

The role of natural killer cells in blocking fibrotic disease is well documented in the liver, and recent publications provide some evidence that they might have similar anti-fibrotic functions in the lungs [10], [26], [27]. NK cells are thought to protect against fibrosis through two different mechanisms: 1) by releasing anti-fibrotic IFN-γ or, 2) by directly killing collagen producing fibroblasts. In fibrotic lungs, NK cells are reported to be active participants in an early stage IFN-γ burst, which is a characteristic of the inflammatory phase (days 1–10) post-bleomycin injection^10, 19, 20^. Similar to their functional capabilities in liver fibrosis, NK cells may also dampen fibrosis during the fibrotic phase (days 10–21), by killing activated fibroblasts [6], [10], [17], [26], [27]. Thus, the anti-fibrotic effects associated with NK cells have the capacity to impact the different pathophysiological phases of BIPF.

To test whether NK cells provide their potential anti-fibrotic effects during the initial inflammatory phase or during the subsequent fibrotic phase of BIPF, we depleted NK cells during each phase. Although NK cells were significantly depleted compared to control sera control in both treatment modes, the diminished numbers did not affect the development of fibrosis as measured by collagen concentration and lung deposition. Cytokine concentrations (IFN-γ, IL-17A, and TGF-β) in the BAL fluid and lung homogenates were similarly unaffected.

Several reports suggest a role for NK cells in pulmonary fibrosis [6], [9], [10]. CXCR3-/- mice deficient mice developed less severe pulmonary fibrosis, inflammation, and cytokine levels, which was associated with a deficiency in NK cell migration to the lung and airways. The sensitivity of CXCR3-/- mice to bleomycin is thought to be related to a deficiency in CXCR3+ NK cell homing, which resulted in significantly less IFN-γ levels in BAL fluid and lung [6]. Although the roles of CXCR3 as well as its ligands CXCL10 and CXCL11 are well established in protecting against BIPF, it is not clear if CXCR3+ NK cells are central to this process [28], [29], [30], [31]. In our experiments depletion of NK cells did not result in any changes in IFN-γ levels in either the BAL fluid or in the lung. Since CXCR3 is expressed by a variety of cells that include activated T cells, NK cells and endothelial cells (all involved in BIPF), the decrease in IFN-γ levels observed in CXCR3-/- mice may be also due to other CXCR3+ IFN-γ producing cells, likely T cells, which are significantly more abundant than NK cells throughout the disease (Fig. 1, 3). Hence, because 1) NK cells represent such a small percentage of the total airway-infiltrating leukocytes, 2) many leukocytes can produce IFN-γ, and 3) depletion of NK cells does not result in any measurable difference in BAL or lung IFN-γ levels, our data suggest that the contribution of NK cells to the overall IFN-γ concentration in the lungs during BIPF is minimal. Furthermore, the role of IFN-γ as a major anti-fibrotic cytokine during pulmonary fibrosis is becoming increasingly controversial. The literature is quite contradictory concerning the role of IFN-γ, since numerous reports demonstrate that mice deficient for IFN-γ develop less severe fibrosis, suggesting a pathological rather than protective role for IFN-γ [32], [33], [34]. The most significant study demonstrating a lack of a protective role for IFN-γ in pulmonary fibrosis is the result of the INSPIRE clinical trial, which concluded that IFN-γ treatment in patients with idiopathic pulmonary fibrosis had no therapeutic effect [35]. While the role of IFN-γ in PF remains controversial, our data indicate that whether NK cells are depleted before bleomycin-induced injury, or during the development of fibrosis, lung or airway IFN-γ levels remain unaltered. These data demonstrate that NK cells are likely not a major contributor to IFN-γ in the BIPF model, and therefore are likely not involved in possible IFN-γ dependent anti-fibrotic pathways.

NKT cells were reported to protect against fibrosis by releasing IFN-γ [9]. Furthermore, mice treated with anti-NK1.1 antibody, which depletes both NKT cells and NK cells, resulted in worse fibrosis in the BIPF model [20], [36]. Anti-asialo GM1 selectively depletes NK cells and basophils but spares NKT cells, and according to the literature basophils are not involved in BIPF or clinical pulmonary fibrosis [12], [16]. Therefore, since NK cell specific depletion by anti-asialo GM1 does not change either IFN-γ levels or fibrosis, and depletion of NK cell and NKT-cells by anti-NK1.1 results in significantly worse fibrosis, the aggregate data suggest that NKT cells but not NK cells play a protective role in pulmonary fibrosis. We unexpectedly found fewer macrophages and neutrophils on day 21 in the group of mice pre-treated and treated throughout the course of BIPF with anti-asialo GM1 (Fig. 4). It is possible that anti-asialo GM1 is up-regulated at some point during this disease on the surface of macrophages and neutrophils, therefore triggering some depletion [12]. Alternatively, NK cell-derived mediators (chemoattractants, for example) may be required for maximal neutrophil and macrophage recruitment, accumulation, or retention in the airways during BIPF. Not surprisingly, treatment with anti-asialo GM1 does not result in a 100% depletion of NK cells; therefore we cannot exclude the possibility that the few remaining NK cells may be sufficient to exert their biological functions without detecting a difference in fibrosis or other fibrosis markers (cytokines, weight loss…). However, in other disease models such as liver fibrosis, influenza infection, and pulmonary metastasis that used an anti-asialo GM1 treatment paradigm similar to one we employed, NK cell depletion resulted in dramatic phenotypes [17], [37], [38]. Indeed, while anti-asialo GM1 treatment resulted in similar significant yet incomplete levels of NK cell depletion as achieved in our studies, in other in vivo models this resulted in increased influenza related mortality, liver fibrosis, and pulmonary metastases [17], [37], [38].

As an alternative approach to test whether NK cells have an effect in BIPF, we adoptively transferred unstimulated NK cells into recipients 12 hours before bleomycin injection. We first tracked the distribution and abundance of transferred NK cells during BIPF using allotypic CD45 markers to distinguish donor from recipient cells. Comparing day one to day 21 post-transfer, the percentage of donor NK cells relative to recipient NK cells decreased slightly from 2.1% to 1.0% in the spleen, indicating that ∼50% of the transferred cells survive for the duration of the study (Fig. 9B). Furthermore, the donor cells were recruited into the airways and lung parenchyma during BIPF, indicating that they are properly positioned to exert any possible effects. Kim et. al reported that 0.3 million transferred NKT cells protected against BIPF [20]; in this study we transferred 1 million NK cells per mouse and evaluated fibrosis on day 21 post-bleomycin injection (note that this is double the number of NK cells used in the NK cell tracking study in Fig. 9). There was a significant increase in the number of BAL lymphocytes in the NK cell recipients vs. saline control (Fig. 10B), which likely reflects the added bulk of NK cells to the recruited population in the airways (although not statistically significant, Fig. 10C supports this interpretation). Adoptively transferred NK cells did not protect against lung fibrosis in the bleomycin model; if anything, there was a trend for increased collagen deposition in the lungs in the NK cell recipient mice (Fig. 10D).

Thus our data suggest that NK cells are dispensable for the development of BIPF and are unlikely to play a protective role in regulating lung fibrosis. Finally, NK cell depletion strategies have been proposed to inhibit persistent viral infection [24], [39] as well as to promote graft vs. tumor responses following allogeneic bone marrow cell transplantation [40]. Our data indicate that such strategies would not contribute to the development or exacerbation of pulmonary fibrosis.
